# 

**Published:** 1915-12-18

**Authors:** 


### WILL EACH READER PLEASE NOTE?

In this Special Number the Editor's aim has been to meet the circumstances created by the sorrows of 1915 by suggesting new interests in directions which make for results. The first nine pages begin with a historical reminder, and proceed to a study of the latest and most complete attempt to provide an inspiring workshop planned to encourage principles of efficiency and economy throughout a great modern voluntary hospital. On page 253 we indicate the directions in which we shall try during 1916 to help forward economical administration on new lines. The Editor's hope is to direct the thoughts of his readers to channels and facts which will enable economy to be practised readily and with knowledge.

**Chelsea Hospital for Women, Fulham Road, S.W.** A sum of £33,000 is still required towards the cost of the completion of the new hospital.

**Guy's Hospital, London Bridge, S.E.,** is dependent upon voluntary support to the extent of nearly £30,000 per annum. The Governors therefore have to rely upon the charitable public to maintain the full efficiency of the Institution.

**Great Northern Central Hospital, Holloway, N.**—An urgent appeal is made for this institution, which at the present time is used for both soldiers and civilians.

**King's College Hospital, Denmark Hill, S.E.,** appeals for annual subscriptions and donations to meet current expenses, and contributions towards the reduction of the large debt still remaining on the new buildings.

**Metropolitan Hospital, Kingsland Road, N.E.**—Here are twenty-six wards for wounded soldiers. Donations to this institution, therefore, are a direct contribution to the needs of our fighting forces.

**Mount Vernon Hospital for Consumption, Hampstead, N.W.,** is in great need of annual subscriptions and donations. The treatment of women and children is a special feature.

**Paddington Green Children's Hospital, W.**—New annual subscriptions and donations are urgently needed, as the average ordinary income of the Hospital and Home falls short of the expenditure by about £1,000. The Hospital owes its bankers £1,620.

**Prince of Wales's General Hospital, Tottenham, N.**—Subscriptions and donations are urgently required to liquidate a debt of £8,000.

**Queen Charlotte's Hospital, Marylebone.**—Sailors' and soldiers' wives have received the benefits of this institution free of cost. An appeal is made for contributions to its truly national work.

**Queen's Hospital for Children, Hackney Road, E.** The health of the children is of vital importance to the nation. £4,300 is required for the present year.

**Royal Free Hospital, Gray's Inn Road, W.C.**—The committee are making strenuous efforts to avoid getting into debt, and they urgently need support.

**Royal London Ophthalmic Hospital, Moorfields, E.C.**—Four years in succession the income of this institution has fallen short of expenditure.

**St. Bartholomew's Hospital, West Smithfield, E.C.**—Owing to the great increase in the cost of maintenance, considerable additional support is essential.

**St. George's Hospital, Hyde Park Corner, S.W.**—The secretary appeals for cigarettes, tobacco, games, etc., for the 500 wounded sailors and soldiers who are being treated at the hospital. Contributions to the general funds also are required.

**St. Mary's Hospital, Paddington, W.**—The regular sources of income yield about £15,000 only. A further sum of £15,000, therefore, is required every year.

**Seamen's Hospital, Greenwich, S.E., and Albert Docks.**—Over 350 men of the Royal Navy and Merchant Service are constantly under treatment, and the nation owes much to the courage, endurance, and skill of the British seamen.

**Westminster Hospital, Broad Sanctuary, S.W.**—Since the beginning of the year 405 military patients have been received, and owing to the great advance in the price of supplies an appeal is made for generous support.

(*Continued on p.* 268.)

### SOME GENERAL CHARITIES.

(*Continued from p.* 254.)

**Royal Hospital for Incurables, Putney Heath.**—£35,000 annually to provide a home for incurables above the pauper class, and for those having a home but no means of support, a pension of £20 a year.

**Church Army, Bryanston Street, Marble Arch, W.**—Donations and gifts in kind of every description.

**Church of England Homes for Waifs and Strays' Kennington Road.**—Funds for the feeding, clothing, and training of over 4,600 children.

**Dr. Barnardo's Homes, Stepney Causeway.**—Child life is the nation's greatest asset. General support for the work of these homes.

**London Orphan School, Watford.**—Contributions to replenish the funds, which have suffered considerably owing to the war.

**Metropolitan Convalescent Institution, 14 Victoria Street, S.W.**—£2,500 to liquidate the debt to the bankers.

**National Children's Home and Orphanage, 104-122 City Road, E.C.**—Further support for the fifteen branches, including an emigration and distribution centre at Hamilton, Canada.

**Mental After-Care Association, Church House, Dean's Yard, Westminster.**—Funds to carry on and extend its work. It is the only association of its kind in the United Kingdom.

### Hospital Needs.

(*Continued from page* 254.)

**Belgrave Hospital for Children, Clapham Road, S.W.**—The expenditure for 1915 has exceeded the income. Contributions needed to make good the deficit.

**London Lock Hospital, Harrow Road, W.**—This institution is greatly in need of new annual subscriptions of £5 5s. each.

**National Hospital for Diseases of the Heart, Westmoreland Street, W.**—Contributions towards reducing the debt of £9,000, or subscriptions towards annual upkeep, are urgently needed.

**St. Andrew's Hospital, Dollis Hill, N.W.**—There is a debt of £9,420, the interest on which amounts to close upon £400 per annum, and is a heavy burden to the institution. Help is needed to supply extra beds, lockers, etc., for the wounded soldiers.

**Victoria Hospital for Children, Chelsea, S.W.**—Immediate help is needed to free the hospital from debt.

RATES OF SUBSCRIPTION: Twelve months, 6/6; Six months, 4/-; Three months, 2/-, post free to any address in the United Kingdom. Abroad, Twelve months, 8/8; Six months, 5/-; Three months, 2/6, post free.—Address The Subscription Dept., "THE HOSPITAL," The Hospital Building, 28/29 Southampton Street, Strand, London, W.C.

